# Surrender, disidentification, and reappraisal: a qualitative comparison of psilocybin responders and non-responders in treatment-resistant OCD

**DOI:** 10.3389/fpsyt.2026.1791689

**Published:** 2026-09-01

**Authors:** Gabrielle Agin-Liebes, Sarah Shnayder, Geena Fram, Thomas Cody Swift, Terence Ching, Anastasia Jankovsky, Jamila Hokanson, Christopher Pittenger, Benjamin Kelmendi

**Affiliations:** 1 Department of Psychiatry, Yale University School of Medicine, New Haven, CT, United States; 2 RiverStyx Foundation, Santa Cruz, CA, United States; 3 Eastern Michigan University, Ypsilanti, MI, United States; 4 Department of Psychology, Yale University, New Haven, CT, United States; 5 Center for Brain and Mind Health, Yale University School of Medicine, New Haven, CT, United States; 6 Yale Child Study Center, Yale University School of Medicine, New Haven, CT, United States; 7 Department of Neuroscience, Yale University School of Medicine, New Haven, CT, United States

**Keywords:** clinical trial, integration, mechanism, non-response, obsessive-compulsive disorder, psilocybin, psychedelic, qualitative

## Abstract

**Introduction:**

Psilocybin treatment has shown promise for treatment-resistant obsessive-compulsive disorder (OCD), yet clinical response is variable. Little is known about the subjective experiences associated with divergent outcomes or the psychological processes differentiating responders from non-responders.

**Materials and methods:**

A qualitative case comparison was conducted using semi-structured interviews from six participants enrolled in a randomized controlled trial of single-dose psilocybin with psychological support for treatment-resistant OCD. Participants were purposively selected to contrast three responders with marked symptom improvement and three non-responders with minimal improvement or worsening symptoms. Interviews were conducted approximately 1-2 months post-dosing and analyzed using reflexive thematic analysis.

**Results:**

Four dialectical themes captured key contrasts between responders and non-responders: 1) Surrender vs. Control, 2) Disidentification with OCD and Self-Compassion vs. Overidentification with OCD, 3) Emotional Breakthrough vs. Emotional Containment, and 4) Reappraisal of OCD as Inconsequential vs. Sustained Conviction in OCD. Among responders, surrender during the acute session appeared to enable subsequent psychological shifts. The self was separated from OCD while self-compassion was developed; emotions were experienced directly in the body without the usual cognitive filtering; and OCD-related concerns were spontaneously reappraised as arbitrary and inconsequential. Among non-responders, control was maintained or surrender was experienced as overwhelming; identification with OCD and persistent self-criticism remained; difficult emotions were avoided or accessed without lasting breakthrough; and conviction in OCD’s logic and demands was maintained or intensified.

**Discussion:**

The distinction between intellectual insight and embodied change emerged as central: responders described viscerally felt insights that spontaneously shaped behavior, whereas non-responders articulated understanding without corresponding change in feeling or action. Across themes, surrender during the acute session appeared to enable subsequent shifts in self-concept, emotional processing, and reappraisal of OCD as inconsequential. Therapeutic benefit may depend on the quality of psychological engagement—particularly the capacity to surrender, tolerate intense emotion, and translate insight into behavioral change—rather than experiential intensity alone. The distinction between intellectual insight and embodied change emerged as central to understanding differential response.

## 1 Introduction

Obsessive–compulsive disorder (OCD) is a chronic psychiatric condition characterized by intrusive obsessions and compulsive behaviors (1). While standard treatments such as selective serotonin reuptake inhibitors (SSRIs) and cognitive-behavioral therapy (CBT) benefit many patients, a substantial subset of patients remain treatment-resistant, experiencing persistent and debilitating symptoms despite multiple interventions (2). For these refractory cases, novel approaches warrant investigation.

Psilocybin, a serotonergic psychedelic, has demonstrated safety and efficacy across depression, addiction, and anxiety disorders (3–6). Emerging evidence suggests that psilocybin may also hold therapeutic potential for OCD treatment. Until recently, the literature was limited to small trials and case reports. The first modern investigation found that all nine participants experienced acute reductions in obsessive-compulsive symptoms ranging from 23–100% within 24 hours; long-term benefit was not systematically explored (7). A more recent open-label study (8) similarly reported moderate-to-large improvements in compulsive behaviors one week after a single psilocybin dose, which diminished by one-month post-treatment.

Our group conducted a randomized, placebo-controlled trial of psilocybin with psychological support for treatment-resistant OCD (9). This trial administered a single moderate dose of psilocybin (0.25 mg/kg) with non-directive psychological support to 28 participants with treatment-resistant OCD; niacin served as active placebo, and participants randomized to placebo were offered open-label psilocybin following unblinding. At the primary endpoint of 48 hours, the psilocybin group showed significant reductions in OCD symptoms compared to placebo, with approximately half of participants demonstrating clinically meaningful improvement by one-week post-dosing. Response was heterogeneous: while some participants experienced rapid symptom relief, others showed minimal or no change. These preliminary findings point toward psilocybin’s potential safety and efficacy for OCD, while underscoring the substantial variability in individual response that remains poorly understood. A recent randomized clinical trial of repeated psilocybin dosing for OCD similarly found that psilocybin was generally well tolerated and associated with reductions in OCD severity, further supporting clinical interest in psilocybin as a potential intervention for OCD while underscoring the need for larger trials (10).

Additional analyses from our group have begun to explore factors underlying this heterogeneity. An interpretative phenomenological analysis of interviews with 12 participants from the same parent trial identified transdiagnostic processes—including surrender, emotional openness, and self-compassion—as central to therapeutic change, suggesting that symptom reduction may depend less on the intensity of the psychedelic experience and more on the quality of emotional engagement it enables (11). Yet clinical response remains variable, and beyond symptom scores, little is known about how individuals actually experience psilocybin-assisted treatment in OCD. While qualitative studies have explored psychedelic therapy in depression and addiction (12–15), OCD has received limited attention (11), and no published work has contrasted the subjective experiences of treatment responders and non-responders within psilocybin therapy for any condition.

The present qualitative case-comparison addresses this gap by examining phenomenological processes during dosing and integration, and how these differed between responders and non-responders—offering insight into how psilocybin may facilitate or fail to facilitate therapeutic change in OCD.

## 2 Materials and methods

### 2.1 Participants

Participants were drawn from a recent randomized, double-blind, active placebo–controlled clinical trial of psilocybin-assisted therapy for treatment-resistant obsessive–compulsive disorder (OCD) conducted at Yale University (9) (NCT03356483). The parent phase 2 trial enrolled 28 adults (ages 18–65) with a primary DSM-5 diagnosis of OCD confirmed by structured clinical interview (M.I.N.I. 7.0.2) (16). All had moderate-to-severe OCD as assessed by the Yale-Brown Obsessive -Compulsive Scale (Y-BOCS ≥ 19) (17) and documented treatment resistance, defined as inadequate response to at least two serotonin reuptake inhibitor trials and, for most participants, prior CBT with exposure and response prevention (ERP). Mean illness duration was approximately 20 years, with average onset in early adolescence (mean = 14 years). Participants were medically stable, not taking psychotropic medication (≥ 14-day washout), and had no history of bipolar or psychotic disorders, active substance use, or suicidal intent.

Participants were randomized to receive a single dose of either psilocybin (0.25 mg/kg) or niacin/placebo (250 mg), administered with standardized, unstructured, non-directive support in a controlled setting. As part of study enrollment, participants were admitted to an inpatient research unit two days before through one day after the dosing session to ensure consistent monitoring. Following unblinding at 48 hours (independent raters remained blinded through week 1), participants randomized to the psilocybin condition continued with follow-up assessments at weeks 1, 2, 4, 8, and 12. Participants initially randomized to niacin/placebo were offered open-label psilocybin after completing week 1 assessments, and all participants elected to receive it.

The present qualitative sub-study focused on six participants (N = 6) purposively selected from the larger trial sample to represent contrasting clinical outcomes. Three responders showed marked improvement (46%, 58%, and 68% Y-BOCS reductions) between baseline and the week 4 time point; three non-responders showed no sustained improvement or symptom worsening (5%, 23%, and 48% Y-BOCS increases). For participants initially randomized to niacin, Y-BOCS change scores reflect outcomes following their open-label psilocybin session, relative to baseline ratings immediately before the session. Selection was based on clear divergence in clinical outcomes and availability of complete qualitative interview data.

Case selection proceeded in two steps. First, participants from the parent trial who had completed a post-psilocybin qualitative interview and had available 1-month post-dosing Y-BOCS outcome data were identified. Second, within this interview-eligible group, we purposively selected cases to maximize contrast between clear responders and clear non-responders. Complete qualitative interview data were defined as an audio-recorded, transcribed post-dosing interview in which the participant completed the core interview domains, including descriptions of the acute psilocybin session, OCD-related material emerging during or after dosing, perceived changes or lack of change following treatment, and reflections on why the intervention did or did not help. Thus, participants were selected not only based on clinical outcome, but also on the availability of sufficiently detailed qualitative material to support within-case and cross-case thematic analysis. Among participants with complete qualitative interview data, the selected responders represented cases with marked symptom improvement, whereas the selected non-responders represented cases with minimal improvement or symptom worsening. The sample should therefore be understood as a purposive case comparison designed to illuminate clinically meaningful contrasts between clear responders and clear non-responders, rather than as an exhaustive analysis of all participants at the most extreme ends of the outcome distribution.

The present sample did not overlap with the 12 participants included in the previously published qualitative analysis from this parent trial (11). Although both studies drew from the same parent clinical trial and used the same semi-structured interview guide, the present analysis examined a distinct set of participants selected to maximize contrast between responders and non-responders. Thus, the current study represents an independent qualitative case comparison rather than a reanalysis of previously reported interview data (11).

All participants provided written informed consent for the clinical trial and qualitative interview. The study was approved by the Yale University Institutional Review Board (HIC# 2000020355).

### 2.2 Interview procedures

Approximately 1-2 months after their psilocybin session (allowing time for initial integration and outcome assessment), participants completed a semi-structured qualitative interview about their experiences. An interviewer (not part of the treatment team) followed a guide covering topics such as the person’s life leading up to the study (context of their OCD and treatments), the subjective experience of their psilocybin session (including any notable visions, emotions, or insights), whether OCD-related phenomena emerged, and reflections on after-effects of the experience (changes in symptoms, outlook, self-concept, etc.). Participants were also invited to discuss any interpretations as to why the treatment did or did not help them. Interviews averaged 60–90 minutes; they were audio-recorded and transcribed verbatim. To encourage openness, the interviewer adopted an empathic, curious stance and reminded participants that there were no right or wrong answers and that honest, even critical feedback about the study was welcome. The interview guide has been published previously (11).

### 2.3 Analytical approach

We conducted a comparative phenomenological case series using reflexive thematic analysis (18). Analysis proceeded abductively (19): prior literature on psychedelic therapy provided sensitizing concepts (e.g., surrender, self-compassion) (12, 20) while codes and themes were developed inductively from the data. Coding was performed using Dedoose qualitative analysis software (21). The coding team comprised three authors (GAL, SS, and GF). The coding team included a clinical psychologist (GAL), a master’s-level clinician in training (SS), and a researcher with a bachelor’s degree in psychology (GF). For each transcript, we generated initial themes while staying close to participants’ own words and concepts. Through an iterative analytic process, we developed a set of higher-order themes that captured recurring aspects of participants’ experiences. The credibility of the analysis was enhanced by investigator triangulation (two analysts independently coded a subset of transcripts and then reconciled interpretations). Any discrepancies in coding were discussed until consensus was reached on theme definitions.

All transcripts were first read in full to develop familiarity with each participant’s narrative and clinical context. Analysts generated initial codes close to participants’ own language, attending to descriptions of the acute psilocybin experience, OCD-related material, emotional and bodily experiences, perceived psychological changes, and post-dosing integration. Codes were then compared within and across cases, with particular attention to differences between participants who showed marked clinical improvement and those who showed minimal improvement or symptom worsening. Through iterative team meetings, lower-order codes were refined into higher-order themes that captured recurring patterns of experience and clinically meaningful contrasts between responders and non-responders.

## 3 Results

The responder and non-responder groups were broadly comparable on key clinical characteristics: all had early-onset OCD (childhood or early adolescence), chronic illness duration (10–30+ years), and overlapping symptom subtypes including contamination, checking, and scrupulosity concerns. The responder group included two cisgender women and one non-binary participant (ages 20s–40s); the non-responder group included three cisgender women (ages 20s–30s).

We identified four overarching themes characterizing the phenomenological experience of psilocybin combined with non-directive support in this OCD sample: 1) Surrender versus Control; 2) Disidentification with OCD and Self-Compassion versus Overidentification with OCD; 3) Emotional Breakthrough versus Emotional Containment; and 4) Reappraisal of OCD as Inconsequential versus Sustained Conviction in OCD. Each theme is presented as a dialectic, reflecting the contrasting experiences of responders and non-responders. The order reflects our analysis: surrender during the acute session appeared to enable subsequent changes in self-concept, emotional processing, and cognitive reappraisal. Participant identifiers note responder (R) or non-responder (NR) status. **Table 1** summarizes participant characteristics; **Table 2** summarizes the four major themes and their contrasting manifestations across groups.

**Table 1:** Participant characteristics and clinical outcomes by responder status.

Responder status	Participant ID	Gender	Age (at study)	Age of OCD onset	Duration of OCD (yrs)	*Y-BOCS % change	Primary OCD subtype(s)
Responder	P1	Female	40s	Childhood	>30	46%	Contamination, Religious/Scrupulosity
Responder	P2	Non-binary	30s	Childhood	~20	58%	Checking, Counting, Moral/Scrupulosity
Responder	P3	Female	20s	Childhood	~20	68%	Mixed (Contamination & Checking)
Non-Responder	P4	Female	20s	Childhood	~10–15	-48%	Checking, Health-related (Body & Food Concerns)
Non-Responder	P5	Female	30s	Childhood	~25	-23%	Contamination, Ordering/Symmetry, Religious/Compulsive Praying
Non-Responder	P6	Female	20s	Early Adolescence	~10–15	-5%	Contamination (Food-related)

Participant ages and durations are presented as ranges to preserve confidentiality. OCD subtypes are summarized from qualitative case materials and clinical documentation. All identifying information has been removed or generalized. *Y-BOCS % Change represents percent reduction in symptoms from baseline to 1-month post-psilocybin dosing session; negative values indicate symptom worsening.

**Table 2:** Summary of themes by responder status.

Theme	Responders	Non-responders
1. Surrender vs. Control	Gradually released control; trusted the process and facilitators; surrender led to emotional relief and insight.	Feared loss of control and remained vigilant or resistant; attempts to surrender felt unsafe or incomplete and did not alter how the experience was managed.
2. Disidentification with OCD and Self-Compassion vs. Overidentification with OCD	Experienced a shift in self-concept marked by disidentification from OCD and increased self-compassion; OCD was no longer experienced as defining the self, allowing greater self-acceptance and agency.	Self-concept remained fused with OCD or was characterized by persistent self-criticism; experiences did not alter how participants related to themselves or soften OCD-driven self-judgment.
3. Emotional Breakthrough vs. Emotional Containment	Emotional experiences revealed a capacity to encounter intense affect directly, altering prior assumptions about how emotion could be tolerated or engaged; emotion functioned as informative rather than something to be avoided, regulated, or managed cognitively.	Emotional experiences were resisted, compartmentalized, or remained within pre-existing comfort zones; emotional access did not alter how emotion was encountered, tolerated, or trusted beyond the session.
4. Reappraisal of OCD as Inconsequential vs. Sustained Conviction in OCD	OCD concerns were spontaneously reappraised as inconsequential; rituals and obsessions lost their felt urgency and appeared arbitrary, meaningless, or “silly” (“None of this matters”).	OCD concerns retained their urgency and power; intellectual understanding did not alter the felt significance of obsessions, and rigid cognitive–behavioral patterns persisted.

### 3.1 Surrender vs. control

Issues of control featured prominently across all participants, consistent with cognitive-behavioral models of OCD that emphasize overestimation of threat, intolerance of uncertainty, and beliefs about the need to control intrusive thoughts — as well as with the phenomenology of psychedelic experience, which is often approached therapeutically through openness, acceptance, and “letting go” (22). A willingness to relinquish control and trust the process appeared to distinguish responders from non-responders.

Responders described initial struggles with the urge to control the psilocybin experience, followed by a pivotal moment of release. P2 (R) wrestled early in the session with the need to manage what was happening, but eventually reached a realization: “It’s all an illusion of control anyway … It’s scary to think that I don’t have control, but the reality is I don’t [have it] anyway.” This insight was accompanied by what they described as a physical and mental “unclenching”—a sense of relaxation as they accepted that many aspects of life were beyond their control. This surrender had lasting effects: weeks later, facing an uncertain medical situation and obsessing for a week over worst-case scenarios, they recalled this insight: “Oh, it doesn’t matter … either way I will find a way”—experiencing calm rather than compulsive worry. They reflected: “I now have a little bit of space and control over how I respond to them [obsessions].”

P3 (R) entered the experience with considerable fear, feeling “very nervous … the uncertainty of what the experience was going to be like.” Her OCD manifested partly through rigid eating rituals—including eating foods in multiples of certain numbers—and a history of disordered eating that she later recognized as connected to her body “not feeling right.” During a frightening early phase, she described “trying very much to just like go with whatever was going to happen” despite the fear. The turning point came when a voice in her experience offered reassurance: “All you have to do is relax here. Nothing’s going to happen to you. The scary part is done now.” This message of safety allowed her to release her grip on control and enter profound calm. Later, when she attempted to direct the experience toward specific content, the voice corrected her: “No, we really don’t, there’s no agenda, we’re just going to like hang out here”—reinforcing that surrender, not management, was what was needed. This shift translated immediately into a changed relationship with bodily intuition: eating wheat thins after surfacing from the experience, she found herself able to simply stop when satisfied—”I had access to this intuition about what I actually wanted for the first time in recent memory.”

P1 (R) similarly described letting go of her need for control, noting that feeling safe during the session—facilitated by the therapeutic environment and presence of trained facilitators—enabled her to relax habitual vigilance. She recalled a pivotal moment: “[The facilitator] said ‘Do you want to hold my hand?’… I was afraid. Once I did, it would let loose, but then I told myself, I’m just going to trust this. And I held his hand and everything started pouring out … I just bawled, cried. It was so much pain. Indescribable.” This act of trust appeared to become the turning point—a surrender that released long-suppressed emotion. The capacity to relinquish control persisted: days later, flying home with intrusive thoughts present, she reported telling herself “I don’t have to pick up these thoughts”—a paradoxical control achieved through accepting lack of control over thought content itself.

For responders, surrender—releasing the fight for control and allowing themselves to be carried by the experience—appeared central to therapeutic breakthroughs, with effects extending beyond the session into daily management of uncertainty and intrusive thoughts.

Non-responders, in contrast, appeared to remain caught in patterns of holding on and resisting the experience. P6 (NR) noticed that “letting go” kept emerging as a theme through imagery and facilitator prompts, yet she reported being unable to fully embrace it. “I feel like when letting go, it’s like an all or nothing thing,” she explained—suggesting that loosening control even slightly felt as destabilizing as losing it entirely. Near the session’s end, at the facilitators’ suggestion, she took a small symbolic risk: removing a comfort object from the protective Ziploc bag she had brought it in—part of her protective rituals around contamination concerns. With 10–15 years of food-related contamination fears, this minor experiment with removing a protective barrier may have represented a significant risk for her, yet she reported experiencing no sense of relief or shift. She later reflected that the letting-go themes felt familiar rather than transformative—she intellectually “knew” control was central to her OCD, but the session did not alter how she related to this understanding emotionally or experientially.

P4 (NR) became actively frightened when she sensed the psilocybin pulling her deeper beyond her control, oscillating between curiosity and fear. She realized she “had some control over how deep [she] went into the experience” and could “choose to try very hard to wake up.” Torn between these impulses, fear ultimately dominated: “Going deeper … was very painful and I didn’t want to,” she said, describing how she repeatedly chose to “come up for air” rather than immerse fully. Notably, she reported an obsessive worry that she “may have accidentally left some aspects of [herself] behind in the experience”—a concern that mapped directly onto her everyday OCD theme of fearing magically leaving parts or aspects of herself in different places and performing rituals to retrieve them.

Across these accounts, responders demonstrated a capacity to relinquish cognitive control, enabling deeper affective processing. Non-responders described experiences in which maintaining control felt necessary for safety, or in which the prospect of surrender generated significant distress. These different relationships to control during the session appeared to be associated with subsequent therapeutic benefit subsequent therapeutic benefit.

### 3.2 Disidentification with OCD and self-compassion vs. overidentification with OCD

Changes in identity in relation to OCD emerged as a central theme. For responders, disidentification from OCD occurred during or immediately after the session—before substantial symptom change—suggesting it facilitated rather than merely reflected improvement. Responders actively separated self from disorder: perceiving OCD as external, recognizing their own preferences as distinct from compulsive demands, and developing self-compassion. Non-responders either maintained fusion between self and OCD or experienced identity shifts without self-compassion, remaining entangled in self-critical frameworks.

P1 (R) recounted a vivid moment of self-recognition that emerged both during and after the session. During the acute experience, she reported “seeing myself in me” while simultaneously perceiving “my OCD was outside of me”—a felt sense of separation between self and disorder. In the days following, as she practiced sitting with difficult emotions and intrusive thoughts without engaging in rumination (“these thoughts are still here but I don’t have to pick them up”), this perception crystallized into a coherent self-image. She described seeing “this raw self without OCD, it was like me, only me. It was just my like, raw, pure me,” and found herself repeatedly returning to this image for comfort. This led to her articulation: “That’s me. That is who I am. Deep down, I’m not all this shit that … I feel has buried me, has suffocated me.” With over 30 years of illness dominated by harm thoughts, relationship OCD, and relentless rumination about whether her life choices and family “felt right,” the recognition of a self-distinct from OCD—a self she described as having been “buried” and “suffocated”—represented a fundamental shift. This shift manifested behaviorally almost immediately: several days after returning home, while out on a run for exercise, she noticed that her mind was “so quiet” that she burst into tears, contrasting sharply with the constant rumination that had characterized even this outlet activity before the study.

P3 (R) demonstrated disidentification through recognizing her agency as distinct from OCD’s demands. She stated: “The ability to choose and to know actually what I want and not just what the OCD wants me to do is mind-blowing to me.” Rather than describing a transformed identity, she emphasized continuity (“I still feel like me”) while gaining clarity about her authentic preferences versus compulsive imperatives. This manifested immediately: walking on sidewalk cracks days after the session, she found “there was the ‘not mattering’ part, and I just didn’t have the ramp up of anxiety … It just like didn’t happen.” She described relearning activities “not in a way that was just dictated by the OCD”—rediscovering ordinary actions like walking or adjusting the radio through her own volition rather than OCD’s organizing influence.

P2 (R) demonstrated both disidentification from OCD and the development of self-compassion. Prior to the session, they reported viewing OCD as inseparable from identity: “I thought it was a very core part of who I am. And what would it mean to let that go?” During the session, they described expecting a transcendent experience—”I wanted to open the door and see God”—but instead encountered themself. They noted that the inner critic they anticipated appeared nonthreatening: “The me that I saw, who was, in theory, this taunting supercharged bully, just looked exactly like me. It wasn’t exaggeratedly evil, or scary … it looked kind of nice, and normal, and soft, and small.” This recognition appeared to shift their self-relation from antagonism to compassion: “That’s just me. I just need to befriend that. I’ve been really mean to me.” Post-session, they reported observing OCD with greater distance: “I can be like, oh, here you are again … but you don’t have as much power over me, you’re not as convincing.” When offered a facilitator’s hand, they held their own instead—a gesture that appeared to embody their realization: “I’m all that I have, and I have to make peace with even this part of my brain that’s really obnoxious and obsessive.”

Across responders, each participant’s experience followed a similar pattern: encountering an authentic self beneath or beyond OCD (P1’s buried self, P3’s renewed access to her own preferences, and P2’s encounter with a softer version of themselves). The shift from identification as a “person with OCD” to a restored sense of self required softening the harsh self-criticism that had characterized their relationship to themselves.

Non-responders presented a contrasting picture. P4 (NR) experienced a shift in self-perception, but one that reinforced rather than loosened her relationship to OCD. She described OCD as “a very core part of the way I perceive the world and my surroundings”—a fusion that the session did not disrupt. She reported a “revelation” of “being something horrible … irredeemable” that she “left the experience not having totally resolved.” Unlike responders who encountered themselves with acceptance, she appeared to encounter a self-defined by moral failure without the self-compassion that might have made this bearable. She explicitly noted the absence: “It didn’t feel like self-forgiveness … almost not even compassionate, felt more like pity. It was just an awareness of the tragedy of that.” Rather than resolving her moral self-scrutiny, the experience appeared to intensify it. In the weeks following, she reported becoming “much more aware of the degree to which my actions, behaviors, and impulses were moral or immoral”—a deepening rather than loosening of her scrupulosity. Her description of the experience as “almost introductory … incomplete” suggests that she may have glimpsed the possibility of a different relationship to herself, but without sufficient self-compassion to translate this encounter into relief.

P6 (NR) similarly appeared to remain fused with OCD, unable to separate self from disorder. The interviewer observed “there’s a little bit of holding on to your life as created by OCD,” which she affirmed. She described “the familiarity that OCD offers you with structuring your life” as a barrier to change. She briefly experimented with releasing a comfort object, describing this as “trying to let go,” but explained her resistance: “if I do that, then I spent all these years not touching certain things or cleaning up, keeping all this stuff separate. And then it was for nothing.” When asked about the possibility that her years of OCD adherence might have been “for naught,” she responded with “resignation, maybe, like, ‘that sucks.’” Her reflections lacked expressions of kindness toward herself for years of struggle. The self-critical framework through which she viewed her life with OCD remained entirely intact.

In summary, disidentification appeared to be an active therapeutic process rather than a consequence of symptom reduction. The temporal sequence was consistent across responders: P1 reported perceptual separation (“my OCD was outside of me”) during the acute session; P3’s recognition of “what I want versus what OCD wants” emerged immediately and preceded behavioral change (testing this on a sidewalk the following day); P2’s encounter with themself—”I opened the door, and I saw myself”—occurred during the session and directly preceded their shift toward self-compassion. In each case, the identity shift appeared to enable rather than follow from symptomatic improvement.

### 3.3 Emotional breakthrough vs. emotional containment

A critical distinction emerged in how participants accessed and experienced emotion during the psilocybin session. Responders described experiences characterized by overwhelming emotional intensity—what P1 called a “flood” of emotion— which was felt to bypass thought and instead were registered directly in the body. Non-responders described varied emotional experiences that lacked the lasting emotional breakthrough that characterized responder sessions.

P1’s (R) experience appears to exemplify this emotional breakthrough. During the session, she described sensing emotion building: “I could feel it coming and I was like, ‘oh my God’, here it comes. It felt like every bit of sadness. Every bit of everything that I’ve ever been buried in me was coming right at me.” When asked to describe the physical sensation, she stated: “It felt completely encompassing my entire body. It was so huge. It literally felt it was coming at me like a freight train and hit me and was in my entire body. I will never be able to fully explain how much it felt. And it felt so incredibly painful.” This appeared to represent not intellectual insight about sadness, but sadness itself—experienced with overwhelming intensity throughout her body. She reported choosing to tolerate rather than resist this experience. When a facilitator offered his hand, she recalled: “I wanted to at that moment, but then I felt afraid. Once I did it would let loose, but then I told myself, I’m just going to trust this. And I held his hand and everything started pouring out.” The result appeared cathartic: “It just felt like I was letting everything out that had ever been in me.” This acute breakthrough appeared to shift her relationship to emotion more broadly. In the days following, she described a new capacity to tolerate difficult feelings: “I just let myself cry, which I normally don’t do … I would cry and then it would feel better.” She reflected that the experience “made me sit with myself and sit with emotions that were uncomfortable that oftentimes I run from.” This shift appeared to extend to positive affect as well: “There’s just a joy that I have internally that I’ve never had … I actually feel the love, I feel joy. I thought I was feeling those things, but it felt not quite right. And now I feel like it’s just whole.”

P2 (R) described what appeared to be a similar shift. They characterized their experience as having “two halves”—the first was “very cerebral negotiation” in which they argued with the experience, and the second seemed to represent a fundamental change: “brain was shut off, feelings were passing directly through my body, and I was feeling things through my body, and for maybe the first time.” They elaborated: “I had felt really, for much of my life, very disconnected from my body, and felt like I didn’t have access to it as an emotional regulator.” The psilocybin experience appeared to dissolve this disconnection: “I always had an intellectual sense of my emotions, but never had felt them in my limbs or skin, and then it felt like that severing had gone away, and I was feeling it all, and that my body is actually maybe a guide.” Describing the lasting impact, they stated: “anger, sadness, and then just a deep calm, as I started to feel things through my body for maybe the first time … I felt like I felt maybe 20 years’ worth of emotions.” For someone with approximately 20 years of OCD characterized by checking, counting, and moral scrupulosity—conditions often organized around cognitive certainty rather than somatic trust—this shift toward embodied emotional experience may represent a fundamental reorientation.

P3 (R) made the distinction explicit: “Nothing about the dosing experience was cognitive, it was like completely embodied and emotional. And I feel like now I have some of that knowledge and understanding and sort of appreciation for emotion that I knew I was supposed to have, but didn’t actually believe at all. Now I believe it more.” She reported having intellectual understanding of emotion’s value but described not experiencing it bodily: “I knew a lot of these things like cognitively, but I couldn’t translate that knowledge into like a gut feeling or like a real true sense in my heart that these things were true.” When encouraged by a facilitator to “don’t try to fight it, just try to go with it,” she reported being able to tolerate the experience, ultimately describing “no qualms about just like expressing whatever the emotion was and the multitude of emotions.” The psilocybin session appeared to provide direct somatic confirmation of what she had previously understood only intellectually.

Non-responders described varied patterns of emotional engagement, but these experiences appeared to lack the key elements observed in responders—the willingness to surrender control, the shift from cognitive to somatic processing, or the capacity to tolerate intensity without resistance.

P4 (NR) experienced emotion but with a quality that seemed to increase rather than resolve distress. She described actively resisting deeper emotional access: “I became aware that I had some control over how deep I went into the experience … going deeper was something that was very painful, not just emotionally, but just very hard, and I didn’t want to.” She reflected on the overwhelming nature of the session: “During the experience it was so difficult. It was so painful. I don’t think I really have been able to stress that enough. It was hard. No one could have prepared me for that … And if anything will stop me from trying it again, it might be that.” Afterward, she reported: “It almost felt like I was too exhausted or in too much shock to really integrate anything … the very verbal internal narrative was something that was still very strong. So I felt like I was going over the factual details of what I had experienced.” Rather than accessing and processing difficult emotion, she appeared to remain caught in cognitive monitoring and distress about the experience itself.

P6 (NR) accessed intense grief during the session, crying extensively about her brother who had died in 2020. She described feeling “stuck and being unable to let go, but needing my brother,” and noted she had never actually grieved him before: “I don’t think I ever even started to actually grieve my brother.” However, rather than surrendering to this grief, she sought escape. She reported wanting to leave the room: “I didn’t want to be there anymore. I wanted to be in a safer space. Specifically, I wanted to be in my car—that’s a very safe space for me.” The sensory experience became overwhelming: “I took off the blindfold and the headphones because it was just too much.” She also expressed discomfort with experiencing the psilocybin while therapists were not sharing the experience. When asked whether the experience provided new perspective, she stated: “Also, not really … it wasn’t a totally new revelation.”

P5 (NR) appeared to demonstrate a different pattern—not resistance to emotion, but an absence of challenging emotional engagement altogether. Unlike P4 and P6, she did not describe struggling against the experience. Instead, her session was characterized by calm and relaxation: “the most deep relaxation and feeling of tranquility, probably that I’ve ever felt … it almost felt like being in a warm bath. It was just so relaxing and lovely.” While she reported some bittersweet feelings, “all the emotions I felt okay.” This ease may reflect emotional containment rather than emotional breakthrough; her experience appeared to remain within comfortable affective territory. The psilocybin session was experienced as pleasant and soothing but did not appear to involve the sustained engagement with challenging emotional material that responders described encountering and moving through.

Across non-responders, emotional experiences did not appear to alter how emotion was encountered, tolerated, or trusted beyond the session itself; emotions were either avoided, contained, or experienced within pre-existing comfort zones.

### 3.4 Reappraisal of OCD as inconsequential vs. sustained conviction in OCD

Responders consistently described their OCD rules and rituals as suddenly appearing arbitrary or without authority both during and after the psilocybin sessions. This shift is clinically noteworthy: the intense, compelling quality of obsessions and compulsions—the sense that one *must* perform rituals or cannot dismiss intrusive thoughts—is central to what maintains the disorder. P3 (R) articulated this most directly through the realization that “it doesn’t matter”—a phrase she repeated during her session that appeared to become her method for disengaging from compulsions. Her elaborate rule systems around numbers, stepping patterns, and eating rituals had previously organized her daily life with rigid authority. The psilocybin experience seemed to reveal these rules as arbitrary constructions without inherent power. When obsessional urges arose, she reported being able to recognize their inconsequentiality: “I’m able to look at it and be like, no, we don’t need to do that.” This reappraisal appeared to have immediate practical effects. She characterized applying the “it doesn’t matter” reframe as “doing the exposures on like easy mode”—suggesting that the mental effort typically required to resist compulsions had been replaced by genuine ease.

P1 (R) described a similar revelation about the arbitrariness of her obsessional patterns. Her decades of rumination suddenly struck her as senseless: “I remember thinking… ‘why have I been ruminating my whole life? …This is so ridiculous.’” This appeared to be not merely insight that rumination was unhelpful—she had known that cognitively for years. Rather, the patterns themselves seemed to appear fundamentally arbitrary and without justification: “Like all these things just seemed that were so intense outside of this experience. I was able to look at everything very rationally and it just all made very, it’s simple sense.” The mental habits that had structured her thinking appeared to lose their grip not through effort but through spontaneous recognition of their inconsequentiality. Post-session, she described lasting change: “My mind, it just wasn’t ruminating, it wasn’t. Even when I was running before, it was constantly thinking and it was so wrapped up … I could see for the first time without OCD, like the blinders were taken off.”

P2 (R) described experiencing this shift as recognizing that OCD’s apparent power was illusory: “it’s a choice, it’s always been a choice.” Where they had felt subject to obsessional demands, they now reported seeing active agency. Their practical mantra—”it doesn’t matter”—appeared to allow them to dismiss compulsions by recognizing obsessional thoughts as “just a voice in my head” without inherent authority or consequence.

Non-responders generally described limited shifts in how they related to OCD, even when they articulated new perspectives at an intellectual level. P5 (NR), in particular, appeared able to consider alternative framings of OCD without these translating into a felt change in its perceived authority. She described having “always viewed OCD as an inevitable thing, or like an all-powerful entity that I was powerless to stop or resist.” Although facilitators introduced reframes she found meaningful—”different ways of framing the OCD or looking at it from a different perspective”—these ideas seemed to remain conceptual rather than experientially integrated. When reflecting on changes following the session, she noted that “the content is pretty much the same,” and that she continued to focus on the same concerns as before, suggesting that her underlying conviction in OCD’s importance may have remained largely intact.

P6 (NR) similarly described little change in how she viewed the power of OCD. Although themes of “letting go” emerged repeatedly during the session, she described them as familiar rather than new, noting that it “wasn’t totally new.” Removing a comfort object from its protective bag also did not bring relief or change how she viewed her contamination concerns. The session appeared to reinforce her existing intellectual understanding of her rigidity without changing her belief that OCD’s rules were necessary.

Interestingly, P4 (NR) appeared to experience a strengthening rather than a loosening of conviction following psilocybin. She noted that her OCD “has always felt very rational to me,” and this perception did not seem to shift during the experience. Instead of describing a change in perspective, she reported becoming “much more aware of the degree to which my actions, behaviors, and impulses were moral or immoral.” This heightened moral awareness may reflect an intensification of the evaluative framework that already characterized her OCD, rather than a departure from it. She acknowledged the limits of this awareness, stating, “I haven’t yet been able to translate that into more adaptive and positive behaviors.” Although the experience appeared to increase insight within her existing belief system, it did not clearly afford a vantage point outside of it. She described the session as feeling “almost introductory … incomplete,” suggesting that while an alternative perspective may have been sensed, it remained out of reach.

Across these accounts, the key distinction appeared to be not insight itself but whether OCD’s authority had been disrupted. Responders seemed to experience genuine reappraisal: rigid rules lost their power, and obsessional demands became dismissible. Non-responders appeared to maintain their conviction in OCD’s logic and importance—or, in P4’s case, may have found it may have experienced an intensification of it. In this sample, psilocybin’s therapeutic effects appeared linked not just to insight, but to whether participants could step outside their entrenched belief in OCD’s demands.

## 4 Discussion

This qualitative case-comparison examined the phenomenological experiences of psilocybin combined with non-directive support in six participants with treatment-resistant OCD, contrasting three responders with marked clinical improvement and three non-responders with minimal improvement or worsening symptoms. Responders described surrendering control, reconnecting with a self beyond OCD, encountering and tolerating overwhelming emotion, and spontaneously reappraising OCD rules as arbitrary. Non-responders, in contrast, maintained control or found surrender overwhelming, remained identified with OCD or self-criticism, struggled to engage difficult emotion, and maintained or intensified conviction in OCD’s logic.

The order of themes reflects what appears to be a sequential relationship among them. Surrender during the acute session seemed to function as a gateway process enabling subsequent shifts in emotional processing, self-relation, and reappraisal. For P1, trusting the facilitator and taking his hand released an emotional flood, followed by seeing her “raw, pure” self beneath OCD and later experiencing rumination as “so ridiculous.” For P2, the shift from “cerebral negotiation” to somatic processing occurred only after they stopped trying to determine their study condition and accepted uncertainty, enabling an encounter with a softer version of themself and the recognition that “it’s always been a choice.” Among non-responders, difficulty surrendering appeared to limit downstream shifts: P4’s fear-driven resistance to going deeper constrained emotional processing, while P6’s desire to escape to her car interrupted grief before it could produce lasting change. Rather than four independent dimensions of response, these themes may represent a temporal cascade in which surrender is a rate-limiting step.

Both responders and non-responders reported gaining insights during their psilocybin sessions; what appeared to distinguish responders was not insight alone, but an emotional experience that disrupted prior assumptions about how emotion could be encountered, tolerated, or trusted. Across responders, emotional experiences appeared to revise participants’ relationship to emotion—by rendering intense affect tolerable, making previously inaccessible emotion available, or shifting emotion from something managed cognitively to something experienced directly. This pattern aligns with prior work identifying emotional breakthrough as a key mediator of enduring psychological change following psychedelic treatment (23). These findings suggest that therapeutic benefit in OCD may depend less on insight itself than on the quality of acute experiential engagement—particularly the capacity to surrender control, tolerate distressing emotion, and experience OCD’s demands as arbitrary rather than imperative. From this perspective, psilocybin may facilitate experiential rather than purely cognitive learning: a shift from cognitive understanding of OCD to a felt recognition of its inconsequentiality. Such a mechanism may be especially relevant for OCD, a disorder characterized by distrust of internal states and compulsive efforts to achieve a felt sense of “rightness” (24). For some participants, particularly those with long-standing OCD, the emotional intensity of the dosing session may have functioned as an experiential disconfirmation of fears about emotional overwhelm.

The patterns observed in this study connect to established models of OCD. Contemporary conceptualizations emphasize intolerance of uncertainty as a core maintaining factor (25, 26) with individuals engaging in compulsions to achieve certainty or the subjective feeling of “just rightness” (27). The responders’ ability to surrender control during psilocybin—explicitly described as learning to tolerate uncertainty—may represent a critical divergence from non-responders who remained stuck in certainty-seeking patterns. Our findings also resonate with the concept of “not just right experiences,” a phenomenological feature in which individuals experience a somatic sense of incompleteness that can drive compulsive behavior. What distinguished responders was not necessarily the elimination of bodily discomfort, uncertainty, or urges, but a transformation in their relationship to bodily experience. Where they previously engaged in compulsions to achieve “rightness,” post-psilocybin they described greater trust in spontaneous bodily responses: P3 could simply stop eating when satisfied for the first time she could remember, P2 discovered that “my body is actually maybe a guide,” and P1 found that emotions moved through her naturally rather than requiring suppression. This shift—from compulsively pursuing certainty about bodily states to trusting spontaneous somatic signals—suggests that psilocybin may help restore trust in one’s own bodily experience, reducing reliance on ritualistic reassurance. For OCD presentations organized around somatic doubt, “just right” sensations, or distrust of internal signals, this restoration may constitute a particularly relevant mechanism.

The distinction between reappraisal and sustained conviction connects to literature on overvalued ideation and insight in OCD. Early researchers proposed that obsessional beliefs vary along a continuum of conviction strength (28), with higher conviction predicting poorer treatment response (29, 30). In our sample, responders reported experiencing a spontaneous collapse of conviction in the validity of OCD rules: beliefs and imperatives that had previously seemed rational and important suddenly appeared arbitrary and dismissible. This shift might be understood as OCD becoming more ego-dystonic: where obsessional concerns had previously felt rational and self-consistent, they came to seem foreign and arbitrary. This differs from cognitive therapy’s typical approach of challenging belief accuracy; responders did not conclude their fears were unlikely but rather that OCD’s rules and imperatives no longer warranted engagement. The phrase “it doesn’t matter,” used independently by both P3 and P2, captures this shift from engagement to non-engagement. Non-responders, by contrast, maintained a more ego-syntonic relationship with their OCD, experiencing their concerns as logical rather than intrusive. P4’s observation that her OCD “has always felt very rational to me”—a belief the experience did not loosen—exemplifies this pattern. Her subsequent intensification of moral scrutiny suggests that psilocybin psilocybin without an accompanying shift shift in the perceived validity of obsessional concerns may reinforce rather than disrupt OCD-related frameworks. Findings from ayahuasca research suggest that this spontaneous conviction shift may be a broader feature of serotonergic psychedelics: one study found that acute experiences of cognitive reappraisal during ceremonial ayahuasca—characterized by accessing challenging psychological content and arriving at resolved perspectives—were the strongest predictors of lasting increases in psychological flexibility (31), suggesting these substances may facilitate a qualitatively different process than the gradual belief modification typical of cognitive therapy. Additionally, this quality of change differed from the effortful, incremental learning typical of exposure therapy (32). Responders described capacities that appeared suddenly accessible rather than gradually acquired: P3 walked on sidewalk cracks without the anticipated “ramp up of anxiety,” and P1 found her mind “so quiet” that she “burst into tears” from joy. This pattern suggests that psilocybin may operate less through gradual habituation than through a sudden reorganization in which prior struggles lose their urgency.

Our findings align with and extend recent qualitative work from our group. In a prior interpretative phenomenological analysis, processes including surrender, emotional openness, and self-compassion were identified as central to participants’ post-dosing experiences (11). The present analysis examined a distinct, non-overlapping sample of six participants from the same parent trial and was designed to address a different analytic question: how subjective experiences differed between clear responders and non-responders. Although several constructs overlap across studies, the present analysis reframes them as dialectical contrasts linked to divergent clinical outcomes: surrender versus control, emotional breakthrough versus emotional containment, and disidentification with self-compassion versus overidentification with OCD. The present study also identifies reappraisal of OCD as inconsequential versus sustained conviction in OCD as an additional responder/non-responder contrast not emphasized in the prior analysis. Thus, these findings suggest that therapeutic benefit may depend not simply on whether participants reported insight, emotional intensity, or self-compassion, but on whether these experiences disrupted OCD’s perceived authority and translated into changes in self-relation, emotional tolerance, and behavior.

Responder narratives suggest that preparation protocols may benefit from explicitly addressing control-relinquishing and psychological flexibility. The adaptive processes observed among responders—surrendering control, encountering and tolerating overwhelming emotion, disidentifying from OCD with self-compassion, and reappraising OCD’s authority—overlap conceptually with Acceptance and Commitment Therapy (ACT) mechanisms for OCD, including cognitive defusion, acceptance, and psychological flexibility (33). However, unlike ACT, which typically cultivates these capacities through repeated practice, responders appeared to access them rapidly, as though barriers to existing capacities had been removed. The absence of such processes among non-responders raises the possibility that brief ACT-informed preparation could enhance engagement with the psilocybin experience (34). Screening for baseline overvalued ideation or strong perceived validity of obsessional concerns may also help identify patients at risk for adverse responses, while integration protocols may need to attend jointly to disidentification and self-compassion.

## 5 Limitations

Several limitations warrant consideration. Our sample was small (N = 6) and purposively selected to represent clear responder and non-responder phenotypes; while this design maximized contrast, it may not capture the full range of treatment responses, and the experiences of partial responders remain unexplored. Because participants were selected based on both clinical outcome divergence and availability of sufficiently complete qualitative interviews, the sample should be interpreted as a focused case comparison rather than a comprehensive analysis of all participants at the most extreme ends of the outcome distribution. Additionally, interviews conducted 1–2 months post-dosing relied on retrospective accounts that may be subject to recall bias—responders may emphasize experiences consistent with their improvement, while non-responders may reconstruct their experiences in ways that explain their lack of change.

Our analysis also focused on psychological processes without integration of neuroimaging data collected as part of the parent trial (9). The mechanistic inferences we draw therefore remain speculative; future analyses integrating phenomenological and neurobiological findings could test whether the psychological differences we observed correspond to measurable differences in brain state. Finally, we cannot determine whether the processes distinguishing responders from non-responders—such as the capacity for surrender—are causes of differential response, consequences of pre-existing traits, or epiphenomena of unmeasured variables. Longitudinal designs tracking psychological processes before, during, and after treatment could help disentangle these possibilities.

## 6 Conclusions

This qualitative case-comparison illuminated divergent phenomenological pathways distinguishing psilocybin responders from non-responders in treatment-resistant OCD. Responders surrendered control, reconnected with an authentic self while developing self-compassion, tolerated overwhelming emotion somatically, and experienced spontaneous reappraisal of OCD’s authority. Non-responders maintained control or found surrender intolerable, remained identified with OCD alongside persistent self-criticism, avoided or could not tolerate difficult emotions, and maintained conviction in OCD’s logic.

These findings suggest that integrating psilocybin with preparation explicitly targeting the capacity for surrender, self-compassion, and tolerance of uncertainty may warrant further investigation, and that screening for baseline conviction strength may help identify patients at risk for adverse intensification of obsessional frameworks rather than their disruption. More broadly, these findings raise a question that extends beyond OCD: whether psychedelic therapy works not by producing insight, but by producing the conditions under which insight becomes embodied—felt in the body, trusted, and spontaneously translated into changed behavior.

## Data Availability

De-identified data supporting the findings of this study may be made available by the corresponding author upon reasonable request, subject to applicable institutional and IRB requirements and protections for participant confidentiality.
